# Can Coronary Artery Involvement in Kawasaki Disease be Predicted?

**DOI:** 10.3390/diagnostics3020232

**Published:** 2013-03-26

**Authors:** Sunil J. Ghelani, Neha S. Kwatra, Christopher F. Spurney

**Affiliations:** 1 Division of Cardiology, Children’s National Medical Center, Washington, DC 20010, USA; E-Mail: sunil.ghelani@cardio.chboston.org; 2 Department of Diagnostic Imaging and Radiology, Children’s National Medical Center, Washington, DC 20010, USA; E-Mail: nskwatra@childrensnational.org

**Keywords:** Kawasaki disease, vasculitis, muco-cutaneous lymph node syndrome

## Abstract

*Background*: Coronary artery involvement is seen in approximately 15–20% of children with Kawasaki disease. There is conflicting literature regarding the clinical and laboratory findings associated with coronary artery involvement. In this retrospective study, we attempt identification of predictive factors for coronary artery involvement at our institute and review the existing literature. *Methods and results*: A review of 203 patients (65% males) with Kawasaki disease was performed, of whom 33 (16.3%) had coronary artery involvement. High erythrocyte sedimentation rate, high platelet count, low hematocrit, low albumin levels, and refractory Kawasaki disease showed significant association with coronary artery involvement. High erythrocyte sedimentation rate and refractory Kawasaki disease were found to be independent predictors of coronary artery involvement. Review of literature suggested a wide range of coronary involvement (<5% to >60%), and highly conflicting clinical and laboratory associations. *Conclusion*: It remains difficult to accurately determine risk of coronary artery involvement, although some laboratory markers may provide information that is helpful for parental counseling and clinical follow up. Future identification of novel biomarkers and host predispositions may further our understanding of coronary artery risks and help personalize therapy for Kawasaki disease.

## 1. Introduction

Coronary artery involvement is the most important complication of Kawasaki disease and is seen in approximately 15–20% of affected [1,2]. Past efforts at using clinical and laboratory parameters to predict coronary artery involvement in Kawasaki disease yielded inconsistent results. Extremes of age, longer duration of fever, higher white blood cell count, lower or higher platelet count, higher C-reactive protein, low hematocrit, and low serum albumin have been reported to be associated with coronary artery involvement in Kawasaki disease [2,3]. A number of scoring systems have attempted to guide decision-making for pharmacotherapy of Kawasaki disease based on risk of coronary artery involvement. These have however not been widely adopted due to complex nature and/or low accuracy [4,5,6]. Because of the imperfect performance of scoring systems, the 2004 American Heart Association guidelines recommend that all patients diagnosed with Kawasaki disease be treated with intravenous immunoglobulin [7]. 

Published literature also reports a wide range of coronary artery involvement rate in Kawasaki disease from less than 5% to over 60% [5,8]. Much of this variability stems from the varying definitions of coronary artery involvement [9]. The first widely used definition of coronary artery involvement in Kawasaki disease was proposed by the Japanese Ministry of Health. It classified coronary arteries as abnormal if the internal lumen diameter was >3 mm in children <5 years old or >4 mm in children ≥5 years old; if the internal diameter of a segment measured ≥1.5 times that of an adjacent segment; or if the coronary lumen was clearly irregular [10]. Coronary artery that appeared dilated on echocardiography but failed to meet these criteria were subjectively called ectatic prior to publication of body surface area adjusted z-scores that are now commonly used [11,12]. It has been shown that use of coronary artery z-scores can identify lesions that may have been previously misclassified as normal [9]. In one large report of over 2,500 patients in the US, the proportion of patients with coronary artery aneurysms increased from 10.0% in 1994 to 17.8% in 2003 [13]. Although the initial pharmacological treatment may not differ based on presence or absence of mild coronary involvement, an accurate definition of coronary involvement is important for the purpose of patient counseling and follow up.

In the present study we retrospectively analyze data from 203 children with Kawasaki disease at our institute for coronary artery involvement and attempt identification of its predictive clinical and laboratory parameters. We also present a pertinent review of existing literature.

## 2. Materials and Methods

Data used were collected in conjunction with a study of the impact of the 2004 American Heart Association guidelines on Kawasaki disease [14]. A retrospective review of medical records was performed for patients with Kawasaki disease (International Classification of Diseases 9th revision code 446.1) between July 2000 and June 2002, and between July 2007 and June 2009. Patients were included only if they had adequate medical records for review and received at least one dose of intravenous immunoglobulin. The study was approved by the institutional review board. Patients’ age, sex, days of fever at diagnosis, presence or absence of each of the principal clinical criteria (rash, conjunctivitis, extremity changes, oral changes, and cervical lymphadenopathy) and laboratory values were collected. Patients with <4 principal clinical criteria were classified as having incomplete Kawasaki disease. All laboratory values were collected before the administration of intravenous immunoglobulin and included white blood cell count, hematocrit, platelet count, C-reactive protein, erythrocyte sedimentation rate, albumin, alanine aminotransferase, and urine leukocyte count. If more than one lab result was available prior to intravenous immunoglobulin administration, the most abnormal value was recorded. Patients were considered to have coronary artery abnormalities due to Kawasaki disease if either any coronary artery segment had a z-score of ≥2.5, adjusted for body surface area, or if they met the Japanese Ministry of Health criteria for coronary artery abnormality. Z-scores were derived for the left main, right proximal, and proximal left anterior descending coronary arteries using equations previously published from our echocardiography database [12]. Refractory Kawasaki disease was defined as persistence of fever after one dose of intravenous immunoglobulin and administration of additional intravenous immunoglobulin or use of corticosteroids or TNF-alpha blockers. We did not use a specific time frame for fever persistence after intravenous immunoglobulin and instead left it to the discretion of the treating physician.

Statistical analysis: Continuous variables were summarized as mean ± standard deviations when normally distributed and as medians and interquartile ranges when nonparametric. Normality of the data was assessed using Shapiro-Wilk test and visual inspection of histograms. Fisher’s exact test and chi-square test were used for comparing proportions as appropriate. Univariable analyses were performed using unpaired t test or Mann Whitney U test to determine whether duration of fever, age, white blood cell count, hematocrit, platelet count, C-reactive protein, erythrocyte sedimentation rate, albumin, alanine aminotransferase or urine white cell count discriminated between patients with and without coronary artery involvement. To identify independent predictors, binary logistic regression models were constructed using the laboratory variables that had been selected by univariable analysis. The discriminatory capacity of the regression model was assessed using the area under the receiver-operating-characteristics curve. Type I error was set at 0.05. All calculations were performed using SPSS Statistics 17 for Windows (IBM Corporation, Armonk, NY, USA) or Open Office Calc (openoffice.org v3.3.0 Oracle Inc., Redwood City, CA, USA).

## 3. Results

Of the 203 patients that met inclusion criteria, 65.0% were males. Median age of the study population was 35 months (interquartile range 16–58 months) and duration of fever at diagnosis was 6 days (interquartile range 5–8 days). Coronary involvement was present in 33 (16.3%) patients. Clinical and laboratory parameters in patients with and without coronary artery involvement are presented in Table 1. Patients with coronary artery involvement had significantly higher erythrocyte sedimentation rate and platelet count and lower hematocrit and albumin levels. The results of multivariate binary logistic regression analysis of variables that were significantly different between those with and without coronary artery involvement are shown in Table 2. Having refractory Kawasaki disease and a high erythrocyte sedimentation rate were independently associated with coronary artery involvement. Goodness of fit of the regression model was confirmed with the Hosmer-Lemeshow test (p value = 0.93), which indicated lack of deviation between the model and observed event rate. Area under the curve for predicted probabilities was 0.82 (95% confidence interval 0.735–0.905, p < 0.001). Among laboratory parameters, erythrocyte sedimentation rate had the strongest association with coronary artery involvement and patients with erythrocyte sedimentation rate ≥80 mm (25.3%) had 4.2 times higher odds (95% confidence interval 2.1 to 10.7) of having coronary artery involvement as compared to those with levels below 80 mm/h.

**Table 1:** Comparison of clinical and laboratory parameters between patients with and without coronary artery involvement. Abbreviations: CA, coronary artery; IQR, interquartile range; CRP, C-reactive protein; ESR, erythrocyte sedimentation rate; WBC, white blood cell; SD, standard deviation; ALT, alanine aminotransferase; HPF, high power field; KD, Kawasaki disease (* p value ≤ 0.05 was considered statistically significant).

	CA Involved (N = 33)	CA Normal (N = 170)	p value	
Male, %	75.8	62.9	0.16	
Age, months, median (IQR)	37 (10.5–61)	34.5 (16.8–55.5)	0.98	
Fever, days, median (IQR)	6 (5–9)	6 (5–8)	0.39	
Extremity changes, %	57.6	69.4	0.18	
Rash, %	78.8	87.6	0.18	
Eyes, %	90.9	85.3	0.58	
Oral, %	87.9	77.6	0.18	
Lymphadenopathy, %	57.6	56.5	0.91	
WBC, ×10^3^ per cubic mm, median (IQR)	14.5 (11.1–18.6)	14.2 (10.1–17.3)	0.54	
Hematocrit, %, mean (SD)	29.5 (4.7)	32.0 (3.4)	0.01	*
Platelets, ×10^3^ per cubic mm, median (IQR)	465 (295.5–670.5)	382 (282.3–484.8)	0.05	*
CRP, mg/dL, median (IQR)	7.0 (3.6–16.1)	5.8 (2.6–10.4)	0.31	
ESR, mm/hr, mean (SD)	86.3 (37.8)	57.3 (26.7)	<0.001	*
Albumin, g/dL, mean (SD)	2.7 (0.6)	3 (0.6)	0.02	*
ALT, units/L, median (IQR)	54 (28–87)	39.5 (27–83.5)	0.45	
Urine white cells per HPF, median (IQR)	2 (0–8.5)	3 (0–14.8)	0.72	
Refractory KD, %	60.6	16.5	<0.001	*
Incomplete KD, %	45.9	35.9	0.33	

**Table 2:** Results of multivariate binary regression analysis. Odds ratio favors coronary artery involvement. Abbreviations: ESR, erythrocyte sedimentation rate(* p value ≤ 0.05 was considered statistically significant).

	Odds Ratio	95% Confidence Interval		p value	
Platelets	1.002	1.000	1.005	0.10	
ESR	1.023	1.006	1.041	0.01	*
Hematocrit	0.965	0.830	1.121	0.64	
Albumin	0.670	0.260	1.722	0.41	
Refractory Kawasaki disease	5.270	2.029	13.689	<0.001	*

## 4. Discussion

In the present study, patients with Kawasaki disease and coronary artery involvement had higher erythrocyte sedimentation rate and platelet count and lower hematocrit and albumin levels. A number of attempts have been made to devise predictive instruments for coronary artery involvement in Kawasaki disease. The Harada score developed in Japan was perhaps the most widely used decision-making tool for the use of intravenous immunoglobulin in Kawasaki disease prior to universal treatment. Children who fulfilled 4 of the following criteria and were assessed within 9 days of onset of illness were administered intravenous immunoglobulin: (1) white blood cell count > 12,000/mm^3^; (2) platelet count < 350,000/mm^3^; (3) C-reactive protein > 3+; (4) hematocrit < 35%; (5) albumin < 3.5 g/dL; (6) age ≤ 12 months; and (7) male sex. For children with <4 risk factors but continuing acute symptoms, the risk score was reassessed daily [4]. In the United States, Beiser *et al.* devised a predictive instrument for the development of coronary artery involvement. Risk factors used in their instrument included baseline neutrophil and band counts, hemoglobin concentration, platelet count, and temperature on the day after intravenous immunoglobulin infusion [5]. This tool had the drawback of a low positive predictive value and fell out of use. Asai *et al.* proposed a scoring system to predict coronary artery involvement based on 15 characteristics, each of which was given a score of 0, 1 or 2 [6]. This scoring system could not be widely adopted as the variables included chest radiograph and electrocardiogram findings and serial measurements of labs; tests which are now not routinely performed for Kawasaki disease.

Inflammatory changes in coronary arteries have been demonstrated in Kawasaki disease even in the absence of aneurysms [15]. Early stages of arterial wall inflammation in Kawasaki disease are characterized by edematous dissociation of the smooth muscle cells, endothelial swelling and subendothelial edema; polymorphonuclear cell infiltration then ensues, which in turn sets the stage for infiltration by mononuclear cells and lymphocytes [16,17]. In cases with ectasia or aneurysm formation there is destruction of the internal elastic lamina and eventually fibroblastic proliferation, followed by several weeks of scarring that may lead to stenosis. As coronary aneurysm formation represents severe inflammation, one would logically expect laboratory markers of inflammation to be higher in patients with visible coronary artery involvement. Gao *et al.* have reported association of fibrinogen polymorphisms and high plasma fibrinogen levels with coronary artery involvement in Kawasaki disease [18]. Fibrinogen is a major determinant of erythrocyte sedimentation rate and a high erythrocyte sedimentation rate was found to have strong association with coronary involvement in the present study. Despite the strong association with erythrocyte sedimentation rate, coronary involvement was not associated with high C-reactive protein levels. Although both fibrinogen and C-reactive protein levels rise in inflammation, they are not directly related proteins and this might explain lack of coronary involvement and C-reactive protein levels in the present study. In addition, C-reactive protein was not commonly performed at our center prior to 2002, it being obtained for 109 of the 203 enrolled subjects. Our analysis may have hence lacked adequate power to detect a significant association between C-reactive protein and coronary involvement.

Refractory Kawasaki disease, usually defined as persistent or recrudescent fever ≥36 h after completion of the initial intravenous immunoglobulin infusion, is perhaps a sign of the most severe form of inflammation and was found to have a strong association with coronary artery disease in the present study [7]. Likewise, most other studies that have primarily focused on predicting refractoriness of Kawasaki disease pointed towards a worse overall laboratory profile and a higher rate of coronary artery involvement [19]. Numerous papers have compared abnormal laboratory findings in patients with Kawasaki disease with and without coronary artery involvement. Table 3 presents some of these studies and demonstrates the existing ambiguity in this regard [2,4,5,6,8,13,20,21,22,23,24,25,26,27,28,29,30,31,32,33,34,35,36,37,38,39,40,41]. The finding that is most consistently reported among these studies is a higher duration of fever (11 of 14 studies that reported this variable). In the present study however, we did not find a significantly higher duration of fever at diagnosis, in patients with coronary artery involvement. This difference is probably accounted for by the difference in the timing of intravenous immunoglobulin. Our study group is relatively recent as compared to many of the older studies wherein intravenous immunoglobulin might have been withheld for a longer duration. The present study also did not find an association between coronary artery involvement and younger age (in agreement with 8 of 16 studies that reported ages), male sex (in agreement with 10 of 14 studies that reported sex), higher white blood cell count (in agreement with 14 of 20 studies that reported white blood cell count), higher C-reactive protein level (in agreement with 6 of 13 studies that reported C-reactive protein ) and higher alanine aminotransferase levels (in agreement with 3 of 4 studies that reported alanine aminotransferase). On the other hand, in the present study, coronary artery involvement was associated with a high erythrocyte sedimentation rate (in agreement with 4 of 12 studies that reported erythrocyte sedimentation rate), high platelet count (in agreement with 2 of 15 studies that reported platelet count), low hematocrit/hemoglobin (in agreement with 5 of 15 studies that report this variable) and a low albumin level (in agreement with 9 of 14 studies that report this variable). These findings highlight the lack of a uniform pattern of conventional laboratory changes in Kawasaki disease and need for better biomarkers to predict coronary artery involvement.

Changes in these traditionally studied laboratory values reflect inflammation, vasculitis, endothelial damage resulting in capillary leak, and tissue repair. Nontraditional laboratory markers such as B-type natriuretic peptide levels, beta thymoglobulin and plasma clusterin have also been reported to predict coronary artery involvement but still need to be tested on a larger scale [37,42,43]. Changes in the level of hepcidin, a central modulator of inflammation associated anemia, are known to negatively correlate with hemoglobin levels and are associated with coronary artery involvement in Kawasaki disease [44]. Although the inflammatory marker levels do not seem to reliably identify those that might have coronary artery involvement, they are invariably elevated in patients with Kawasaki disease. This points towards presence of factors other than the severity of inflammation that play a role in determination of coronary artery involvement. In the recent years a number of genetic markers including certain genetic polymorphisms, specific genes, human lymphocyte antigen types, calcium dependent NFAT signaling, caspase-3, and TGF beta genes have been associated with coronary artery involvement in Kawasaki disease [45,46,47,48,49,50,51,52,53,54,55,56,57]. Yamamura *et al.* have recently reported higher risk of coronary involvement in BB blood group genotype [58]. Future studies will need to identify novel markers and host characteristics to supplement clinical and traditional laboratory parameters to guide the clinical decisions about the use of efficacious, but expensive and potentially harmful therapies for Kawasaki disease. Identifying this risk of coronary artery involvement may be of particular importance in those children who continue to have fever despite one or two doses of intravenous immunoglobulin and are considered candidates for newer therapies including TNF-alpha blockers and other biomodulators.

This study is limited by its retrospective nature and relatively small sample size. By retrospective design, it was not possible to record laboratory results on a uniform day of illness. We therefore utilized the most abnormal laboratory result prior to IVIG administration which has the potential to lead to bias due to extreme values. Although we performed a detailed review of literature, we did not do a formal meta-analysis of the question. Given the absence of a detailed national Kawasaki disease database to perform a uniform large-scale analysis, a formal meta-analysis may support these initial findings.

**Table 3:** Variability in reported clinical and laboratory findings associated with coronary artery involvement in Kawasaki disease. Yes indicates presence of association with coronary involvement; No, absence of association; Up and down arrows indicate high or low values of the laboratory parameters; Fever indicates longer duration of fever. In the platelets column, High indicates association of coronary involvement with a higher platelet count and Low indicates association of coronary involvement with a low platelet count; Abbreviations: CAI, coronary artery involvement; KD, Kawasaki disease; iKD, incomplete KD; rKD, refractory KD; ESR, erythrocyte sedimentation rate; WBC, white blood cell; N, Neutrophil count; Plt, platelet count; CRP, C-reactive protein; ALT, alanine aminotransferase; Hct, hematocrit/hemoglobin. * In this study the white blood cell count, platelet count and C-reactive protein significantly increased after intravenous immunoglobulin in patients with KD but levels were similar to those without coronary involvement prior to IVIG, ^#^ In this study the labs were obtained after intravenous immunoglobulin therapy. ^@^ This study is a meta analysis of Chinese literature. ^&^ These two studies do not specify the duration of fever, however report the duration of illness/symptoms prior to IVIG administration. This duration is likely to be same or similar to duration of fever in most cases; however this may not be true for all patients.

Study	n	CAI%	↓Age	Fever	iKD	rKD	Male	↑ESR	↑WBC	↑N	Plt	↑CRP	↑ALT	↓Hct	↓Albumin
Asai *et al.* [6]	102	14.7	Yes	Yes				Yes	Yes			No		No	
Ishihara *et al.* [20]	130	23.8	Yes	Yes			No	No	Yes		No			Yes	Yes
Koren *et al.* [21]	163	15.0	No	Yes			No	No	No		No			No	
Nakano *et al.* [22]	78	20.5	No				No		No		No	Yes		No	No
Nakano *et al.* [23]	22	36.4								Yes					Yes
Ichida *et al.* [25]	110	22.7	Yes	Yes			No	Yes	No		High			No	
Daniels *et al.* [26]	77	12	No	Yes			No	No	No		No			Yes	
Harada *et al.* [4]	258	13.2	Yes				Yes		Yes		Low	Yes		Yes	Yes
Lu *et al.* [27]	70	21							Yes			Yes			
Beiser *et al.* [5]	760	4.3								Yes	Low				
Mori *et al.* [28] * ^&^	193	12.2	No	No			No		No	No	No	Yes			No
Morikawa *et al.* [29]^ #^	451	6.9		Yes					Yes	Yes		Yes		Yes	Yes
Nomura *et al.* [30]	125	15.2							No			No	Yes		
Honkanen *et al.* [32]	344	28.5	Yes	Yes			Yes	No	No		No			Yes	Yes
Belay *et al.* [13]	2,798	12.9	Yes				Yes								
Durongpisitkul *et al.* [34]	432	14.5		Yes		Yes	Yes			Yes					
Kim *et al.* [35]	285	6.7	No	Yes	Yes	Yes	No		No	Yes	No	Yes		No	No
McCrindle *et al.* [2]	190	26	Yes	Yes											Yes
Chaiyarak *et al*. [33]	96	28.9						No							
Kim *et al*. [36]	475	33.5	No	Yes	Yes	Yes		No	No	No	No	No		No	Yes
Kaneko *et al* [37] ^&^	43	14.0	No	No		No	No		No			No			No
Chen *et al.* [38]^ @^	8,330							Yes	No	Yes	High			No	Yes
Tremoulet *et al.* [39]	380	31.6						Yes	Yes						
Caballero-Mora [40]	76	15.7						No	No		No	Yes	No	No	No
Weng *et al.* [41]	216	37.5	No			Yes	No		No	Yes	Low	No	No	No	
Zhang *et al.* [8]	553	63.3	Yes	No	Yes		No	No	No		No	No	No	No	Yes

## 5. Conclusion

Based on proven efficacy and reasonable safety profile of intravenous immunoglobulin, and review of literature, the authors agree with the 2004 American heart association guidelines that all patients with Kawasaki disease should receive therapy with intravenous immunoglobulin irrespective of laboratory changes. It remains difficult to accurately determine risk of coronary artery involvement. Future identification of novel biomarkers and host predispositions may further our understanding of coronary artery risks and help personalize therapy for Kawasaki disease.
